# Association between individual retinal layer thickness and visual acuity in patients with epiretinal membrane: a pilot study

**DOI:** 10.7717/peerj.9481

**Published:** 2020-07-10

**Authors:** Jing Zou, Wei Tan, Wenlong Huang, Kangcheng Liu, Fangling Li, Huizhuo Xu

**Affiliations:** 1 Eye Center of Xiangya Hospital, Central South University, Changsha, Hunan Province, China; 2 Hunan Key Laboratory of Ophthalmology, Changsha, Hunan Province, China

**Keywords:** Retinal layer thickness, Visual acuity, Epiretinal membrane, Foveal region, Parafoveal region, Perifoveal region

## Abstract

**Purpose:**

We investigated the correlation between visual acuity (VA) and individual retinal layer thickness in the foveal, parafoveal, and perifoveal regions of patients with an idiopathic epiretinal membrane (ERM).

**Methods:**

One hundred and five subjects presenting with unilateral idiopathic ERM were included in this study. We segmented each patient’s optical coherence tomography (OCT) image into seven layers and calculated the mean layer thickness in the foveal, parafoveal, and perifoveal regions using the Iowa Reference Algorithm. In 105 patients with ERM, we detected correlations between their macular regions’ individual retinal layer thickness and their best corrected VA. Thirty-one of the 105 patients with ERM underwent vitrectomy and completed six months of follow-up. We then compared the 31 surgical patients’ preoperative and postoperative individual retinal layer thickness in each macular region. Additionally, the association between preoperative individual retinal layer thickness in each macular region and VA six months post-surgery in patients with ≥ two Snellen lines of visual improvement was determined.

**Results:**

Multiple linear regression analysis showed that the inner nuclear layer (INL) thickness in the foveal, parafoveal, and perifoveal region were all associated with VA in the 105 patients (*R*^2^ = 0.344, *P* < 0.001; *R*^2^ = 0.427, *P* < 0.001; and *R*^2^ = 0.340, *P* < 0.001, respectively). Thirty-one surgical patients 6 months post-surgery showed significantly decreased thicknesses (*P* ≤ 0.012) of the foveal INL, inner plexiform layer (IPL), and outer nuclear layer (ONL); the parafoveal retina nerve fiber layer (RNFL), IPL, INL, and ONL; and the perifoveal RNFL, IPL, INL, ganglion cell layer (GCL), outer plexiform layer (OPL), and photoreceptor layer (PRL). We found a weak correlation between postoperative VA and preoperative foveal and perifoveal RNFL thickness (*r* = 0.404 and *r* = 0.359, respectively), and a moderate correlation between postoperative VA and preoperative foveal and parafoveal INL thickness (*r* = 0.529 and *r* = 0.583, respectively) in the 31 surgical patients (*P* ≤ 0.047). The preoperative INL thickness in the foveal, parafoveal, and perifoveal regions showed a moderate to strong correlation (*r* = 0.507, 0.644, and 0.548, respectively), with postoperative VA in patients with ≥ 2 lines of visual improvement (*P* ≤ 0.038).

**Conclusion:**

We detected a correlation between retinal damage and VA in the parafoveal, perifoveal, and foveal regions. Our results suggest that INL thickness in all macular regions may be a prognostic factor for postoperative VA in ERM patients.

## Introduction

An epiretinal membrane (ERM) is one of the primary sources of visual impairment in elderly patients, with an estimated prevalence between 7.9% and 13% according to recent large population studies (Cheung et al., 2017; Ye et al., 2015). An ERM’s clinical manifestation is characterized by reduced visual acuity (VA), increased metamorphopsia, and central vision loss (Chang et al., 2017; Dawson, Shunmugam & Williamson, 2014). A pars plana vitrectomy (PPV) with ERM removal is a standard surgical procedure that can restore the retinal anatomical structure (Naruse, Shimada & Mori, 2017; Tognetto et al., 2019). However, symptoms may persist in some patients after surgery, increasing the interest in identifying predictive factors for postoperative visual outcomes, such as preoperative VA, inner segment/outer segment integrity, central foveal thickness, and separate layer thickness (Garcia-Fernandez et al., 2013; Inoue et al., 2011; Kim et al., 2013).

Individual retinal layer thickness can be measured using spectral domain optical coherence tomography (SD-OCT), analysis software (Terry et al., 2016), or the Iowa Reference Algorithm. This algorithm was used to automatically measure the nine regions determined by the Early Treatment Diabetic Retinopathy Study (ETDRS) grid with high repeatability and reliability (Demirkaya et al., 2013; Garvin et al., 2009; Sohn et al., 2013; Song, Lee & Smiddy, 2016). Although previous studies focused solely on the foveal retina, the roles of the parafoveal and perifoveal retinal regions in ERM pathogenesis have only recently been explored. Arichika, Hangai & Yoshimura (2010) manually measured inner and outer retinal thickness in the foveal, parafoveal, and perifoveal regions to show their connection to VA in ERM patients. Parafoveal inner nuclear layer (INL) thickness was shown to be a possible predictive factor for visual rehabilitation in ERM patients (Kim et al., 2013). Other studies reported that increased retinal perfusion in the foveal, parafoveal, and perifoveal regions after surgery may act as a visual prognosis factor (Chen et al., 2019; Mastropasqua et al., 2019). We inferred that after ERM removal, the macular retinal structure not only changed in the foveal region, but in the parafoveal and perifoveal regions as well. In this study, we used the Iowa Reference Algorithm to measure the thickness of seven individual retinal layers in each of the ERM patients’ macular regions. We ultimately confirmed the correlation between individual retinal layer thickness and pre- and post-surgery VA.

## Materials & Methods

### Subjects

For this retrospective study, we selected a total of 105 eyes from 105 patients diagnosed with a unilateral idiopathic ERM between October 2017 and May 2019. We received approval from the Xiangya Hospital Medical Ethics Committee (No. 201910413), and obtained written informed consent from all patients. All methods in this study followed the Helsinki Declaration and medical codes of conduct.

Our study included eyes with fibrous membranes in front of the macula, which were detected and confirmed using simultaneous fundus and OCT examinations. The exclusion criteria were: (1) secondary macular ERMs caused by retinal detachment or inflammatory disease; (2) severe cataract (Lens Opacities Classification System (LOCS) III Cortical opacity grade 3 or higher) and nuclear opacity (Chylac Jr et al., 1993); (3) high myopia (spherical equivalent of ≥ −6.0 diopters or axial length > 26 mm); (4) previous retinal surgery or trauma; (5) history of hypertension or diabetes; (6) previous history of endoscopic or laser treatment; (7) co-occurrence with retinal or optic nerve disease; and (8) eye inflammation, cornea abnormality, or any other ocular disease that can affect the patient’s vision.

Sixty out of 105 patients underwent vitrectomy for idiopathic ERM removal. We excluded surgical patients if they had any of the following conditions: (1) cataract progression after surgery affecting visual function (LOCS III cortical opacity grade 3 or higher, and nuclear opacity); (2) ERM recurrence after surgery; (3) eye inflammation, cornea abnormality, or any other ocular disease that can affect the patient’s vision after surgery; (4) elevated intraocular pressure > 21 mmHg measured using Goldmann applanation tonometry; and (5) a lack of available postoperative clinical follow-up information after 6 months. Ultimately, we included 31 surgical patients with ERMs.

### Ophthalmic examinations

The following ophthalmic examinations were performed on all patients: VA measurement, intraocular pressure (IOP) measurement, slit lamp microscopy examination, fundus examination using a 90D lens, and SD-OCT scan.

Dr. Huizhuo Xu, a senior surgeon, performed a 23-gauge PPV on the patients requiring surgery. Under retrobulbar anesthesia, the posterior vitreous cortex was removed using triamcinolone acetonide. Subsequently, the ERM was removed and a fovea-sparing inner limiting membrane (ILM) peeling was performed after the ILM was stained with indocyanine green (Liu et al., 2015). All surgical patients underwent ophthalmic reexaminations 1, 3, 6, and 12 months following surgery. This reexamination included VA measurement, IOP measurement, slit lamp microscopy examination, fundus examination with a 90D lens, and SD-OCT scan.

### Optical coherence tomography and layer segmentation

All subjects were OCT scanned using the SD-OCT in the 512 ×  128 mode (Cirrus; Carl Zeiss Meditec Inc, Dublin, CA, USA) which produced 6×6 mm volumetric macular images. We excluded poor-quality scans with a strength index less than 7, scans with misalignment, and decentered scans.

**Figure 1:**
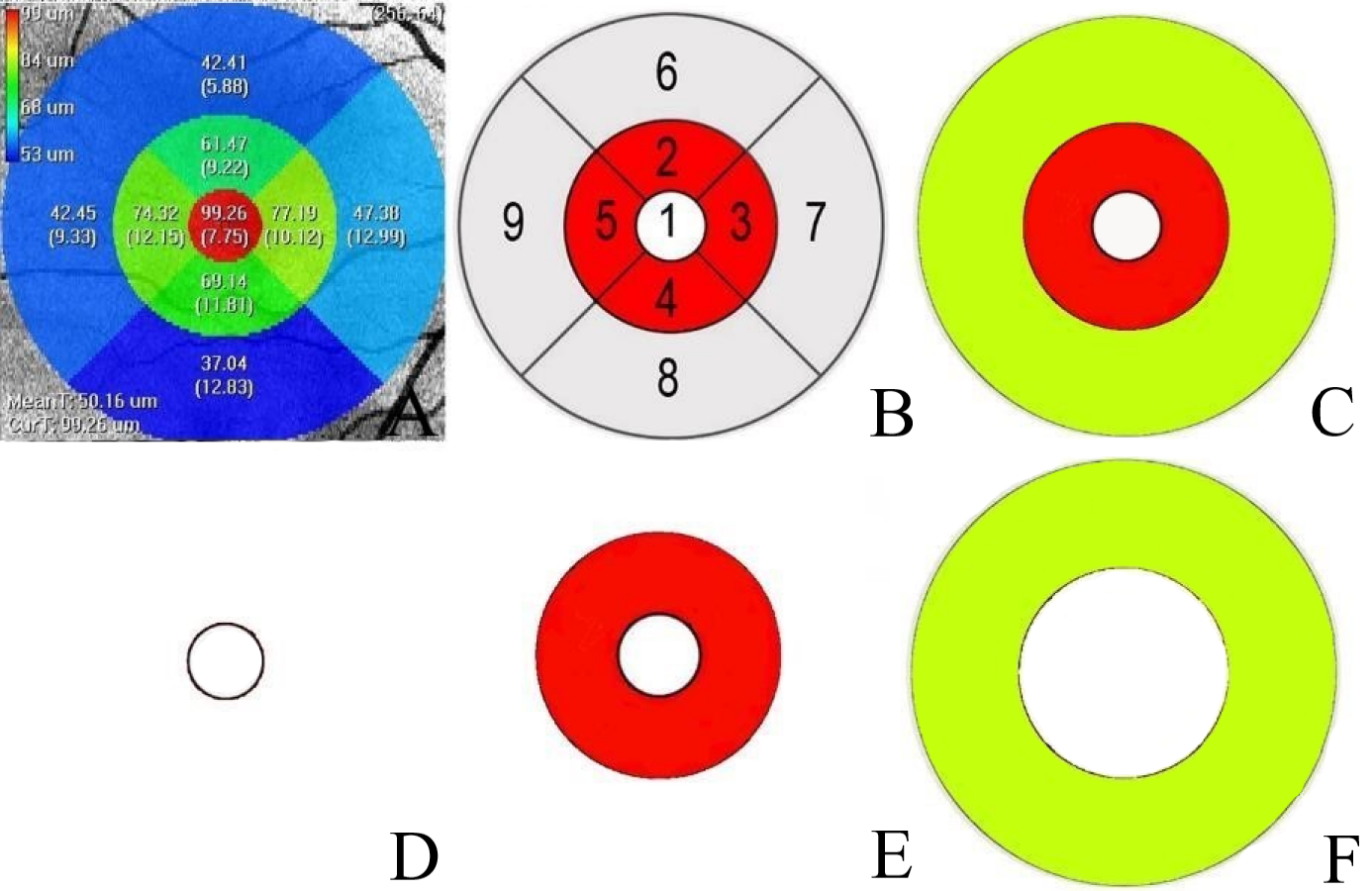
Macular area measurement map. (A) The Iowa Reference Algorithm software divided the image into nine subfields according to the Early Treatment Diabetic Retinopathy Study (ETDRS) grid and measured the macular layer thicknesses in each subfield. (B) Nine subfields numbered according to the ETDRS grid. Area 1′ s measurement was the mean thickness of the foveal region. The mean thickness of the parafoveal region was calculated by averaging the thickness measurements of Areas 2, 3, 4, and 5. The mean thickness of the perifoveal region was calculated by averaging the thickness measurements of Areas 6, 7, 8, and 9. (C) The white circle is the foveal region. The red and yellow regions were calculated separately at different distances from the fovea: red (1–3 mm diameter region) and yellow (3–6 mm diameter region). (D). The white circle with a one mm diameter constituted the foveal region. (E) A ring-type red region with a 1–3 mm diameter constituted the parafoveal region. (F) A ring-type yellow region with a 3–6 mm diameter constituted the perifoveal region.

The Iowa Reference Algorithm (version 3.8) directly segmented the images after reading the input OCT scans. The software divided each patient’s image into nine subfields (segments 1–9) according to the ETDRS grid (Fig. 1A). We calculated the separate thickness values of the nine regions’ individual retinal layers at various distances from the fovea: one mm distance (foveal), 1–3 mm distance (parafoveal), and 3–6 mm distance (perifoveal) (Fig. 1C). The mean parafoveal thickness was calculated by averaging the thicknesses of segments 2 to 5, and the mean perifoveal thickness was obtained by averaging the thicknesses of segments 6 to 9 (Demirkaya et al., 2013) (Fig. 1B).

The Iowa Reference Algorithm automatically segmented the whole retina into layers (retina nerve fiber layer (RNFL), ganglion cell layer (GCL), inner plexiform layer (IPL), INL, outer plexiform layer (OPL), outer nuclear layer (ONL), photoreceptor inner/outer segments (IS/OS), outer segment (OS) and retinal pigment epithelium (RPE), and calculated each individual layer’s thickness. We measured the thickness of the RNFL, GCL, IPL, INL, OPL, ONL, PRL (photoreceptor layer; IS/OS+OS), and the entire retina.

### Statistical analysis

We used the Snellen chart to measure VA, which we then converted to the logarithm of the minimum angle of resolution (LogMAR) for statistical analysis. Statistical analyses were performed using SPSS version 20.0 (SPSS IBM, Inc., Chicago, IL, USA).

We assessed the normal data distribution using the Kolmogorov–Smirnov test. All data were presented as the mean ±  standard deviation for continuous variables. Multiple linear regression analyses were conducted to analyze the relationship between preoperative VA and preoperative individual layer thickness in each macular region of the 105 ERM patients. We established three regression models for predicting preoperative VA based on individual layer thickness in the foveal, parafoveal, and perifoveal regions. Linear regression analysis was used to evaluate the correlation between preoperative VA and INL thickness of each macular region. The comparison between the pre- and postoperative thickness of the individual retinal layers was conducted with a Wilcoxon signed test in the 31 surgical patients. We used a Spearman’s rank correlation coefficient test to analyze the correlation between postoperative VA and preoperative thickness of the individual retinal layers in each macular region. Significance was established when *P* < 0.05.

## Results

Our final sample included 105 eyes with ERM from 105 patients. Thirty-one of the 105 patients who met our criteria were surgical patients. Table 1 summarizes the clinical data of all 105 subjects and the 31 surgical patients, including their age, sex, lens status, and VA.

**Figure 2:**
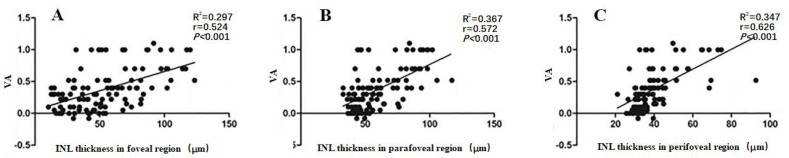
The correlation between VA and the INL thickness in the foveal (A), parafoveal (B) and perifoveal (C) regions in 105 patients with ERM. Abbreviations: inner plexiform layer; INL, VA, visual acuity; LogMAR, logarithm of the minimum angle of resolution. ERM: epiretinal membrane.

Multivariate stepwise regression analyses found a significant correlation between retinal thickness in each macular region and VA in the 105 ERM patients (*P* ≤ 0.013). We estimated VA using three linear predictive models based on the individual layer thickness of the foveal, parafoveal, and perifoveal regions (Table 2). As shown in equation (1), we could predict changes in VA by looking at changes in RNFL (*P* = 0.003) and INL (*P* < 0.001) thickness. The determination coefficient *R*^2^ = 0.344 indicated that 34.4% of the VA variation may have been attributed to the joint variations in foveal RNFL and INL thickness. Equation (2) shows that we could predict VA changes by looking at changes in the INL (*P* < 0.001), OPL (*P* = 0.010), and PRL (*P* = 0.013). The determination coefficient *R*^2^ = 0.427 indicated that 42.7% of the VA variation may have been attributed to joint variations in parafoveal INL, OPL, and PRL thickness. Equtaion (3) shows that we could predict changes in VA by looking at changes in INL (*P* < 0.001) thickness. The determination coefficient *R*^2^ = 0.340 indicated that 34.0% of the VA variation may have been attributed to joint variations in perifoveal INL thickness. All equations revealed that the INL thickness in all macular regions could affect ERM patients’ VA (*P* < 0.001). Additionally, we used a linear regression analysis to assess the correlations between VA and INL thickness. Figure 2 shows the correlations between VA and foveal (*r* = 0.524, *P* < 0.001), parafoveal (*r* = 0.572, *P* < 0.001), and perifoveal (*r* = 0.626, *P* < 0.001) INL thickness, respectively.

**Table 1:** Clinical features and parameters of the participants.

Parameter	105 ERM patients	31 surgical patients
Age (years)	61.85 ± 6.56	64.00 ± 5.90
Male	42 (40%)	19 (61%)
LogMAR	0.38 ± 0.32	0.55 ± 0.30
lens status		
lucent lens	27 (26%)	7 (23%)
Grade I cortical cataract	43 (41%)	13 (42%)
Grade II cortical cataract	35 (33%)	11 (35%)

**Notes.**

Data are presented as the mean ± standard deviation for continuous variables and as frequencies (%) for all other variables.

**Table 2:** Equations for predicting VA (multiple linear regression, *n* = 105).

**Macular** **region**	**Included variables**	**Regression formula**	**Adjusted** ***R***^**2**^	**Residual** **variance**	**significance**
Foveal	Thickness of RNFL and INL (µm)	0.002RNFL+0.005INL+0.046 (1)	0.344	6.835	0.003
Parafoveal	Thickness of INL, OPL, PRL (µm)	0.007INL+0.014OPL+0.016PRL3-1.002 (2)	0.427	5.908	0.013
Perifoveal	Thickness of INL (µm)	0.016INL-0.243 (3)	0.340	6.940	<0.001

**Notes.**

Abbreviations RNFL retinal nerve fiber layer INL inner nuclear layer OPL outer plexiform layer PRL photoreceptor *R*^2^ determination coefficient

We compared the seven individual retinal layer thicknesses in each macular region belonging to the 31 surgical patients. The foveal IPL, INL, and ONL thicknesses were significantly lower than the preoperative thicknesses (*P* < 0.001). The parafoveal RNFL, IPL, INL, and ONL thicknesses dropped off dramatically (*P* < 0.001), and the perifoveal RNFL, IPL, INL, OPL, and PRL also showed a decline (*P* < 0.012) (Table 3). The INL thickness was significantly lower across all macular regions.

**Table 3:** Comparison of the thickness of individual retinal layers in each macular region before and after surgery in 31 surgical ERM patients.

**Retinal Layers**	**Postoperative (µm)**	**Preoperative (µm)**	***P*-value**		**Retinal Layers**	**Postoperative (µm)**	**Preoperative (µm)**	***P*-value**
RNFL					OPL			
foveal	58.17 ± 55.26	76.15 ± 64.52	0.147		foveal	34.01 ± 9.01	34.47 ± 6.44	0.644
parafoveal	38.15 ± 19.21	68.98 ± 42.09	<**0.001**		parafoveal	34.42 ± 5.21	33.93 ± 4.22	0.614
perifoveal	42.81 ± 12.65	62.21 ± 20.05	<**0.001**		perifoveal	28.31 ± 3.06	30.33 ± 3.29	**0.011**
GCL					ONL			
foveal	58.1 ± 23.69	62.69 ± 28.89	0.329		foveal	93.79 ± 38.41	130.9 ± 22.65	<**0.001**
parafoveal	54.17 ± 13.93	58.43 ± 16.08	0.206		parafoveal	87.7 ± 29.48	112.07 ± 24.37	<**0.001**
perifoveal	29.1 ± 6.4	32.34 ± 7.05	**0.012**		perifoveal	76.12 ± 25.66	90.81 ± 20.86	0.065
IPL					PRL			
foveal	29.14 ± 5.66	42.25 ± 13.22	<**0.001**		foveal	38.27 ± 4.91	37.09 ± 6.11	0.272
parafoveal	35.91 ± 4.57	48.94 ± 12.35	<**0.001**		parafoveal	32.25 ± 4.13	33.41 ± 4.57	0.202
perifoveal	35.35 ± 4.81	41.85 ± 7.9	<**0.001**		perifoveal	30.61 ± 3.52	32.71 ± 3.45	**0.008**
INL					Total			
foveal	55.61 ± 22.53	75.56 ± 28.49	<**0.001**		foveal	415.53 ± 86.99	504.93 ± 118.14	<**0.001**
parafoveal	62.92 ± 21.38	75.88 ± 28.47	<**0.001**		parafoveal	392.68 ± 60.25	470.61 ± 94.04	<**0.001**
perifoveal	43.8 ± 12.74	49.58 ± 14.61	**0.002**		perifoveal	328.02 ± 38.66	370.44 ± 59.5	<**0.001**
LogMAR	0.32 ± 0.28	0.55 ± 0.3	**0.001**					
:

**Notes.**

Abbreviations RNFL retina nerve fiber layer GCL ganglion cell layer IPL inner plexiform layer INL inner nuclear layer OPL outer plexiform layer ONL outer nuclear layer PRL photoreceptor layer

**Figure 3:**
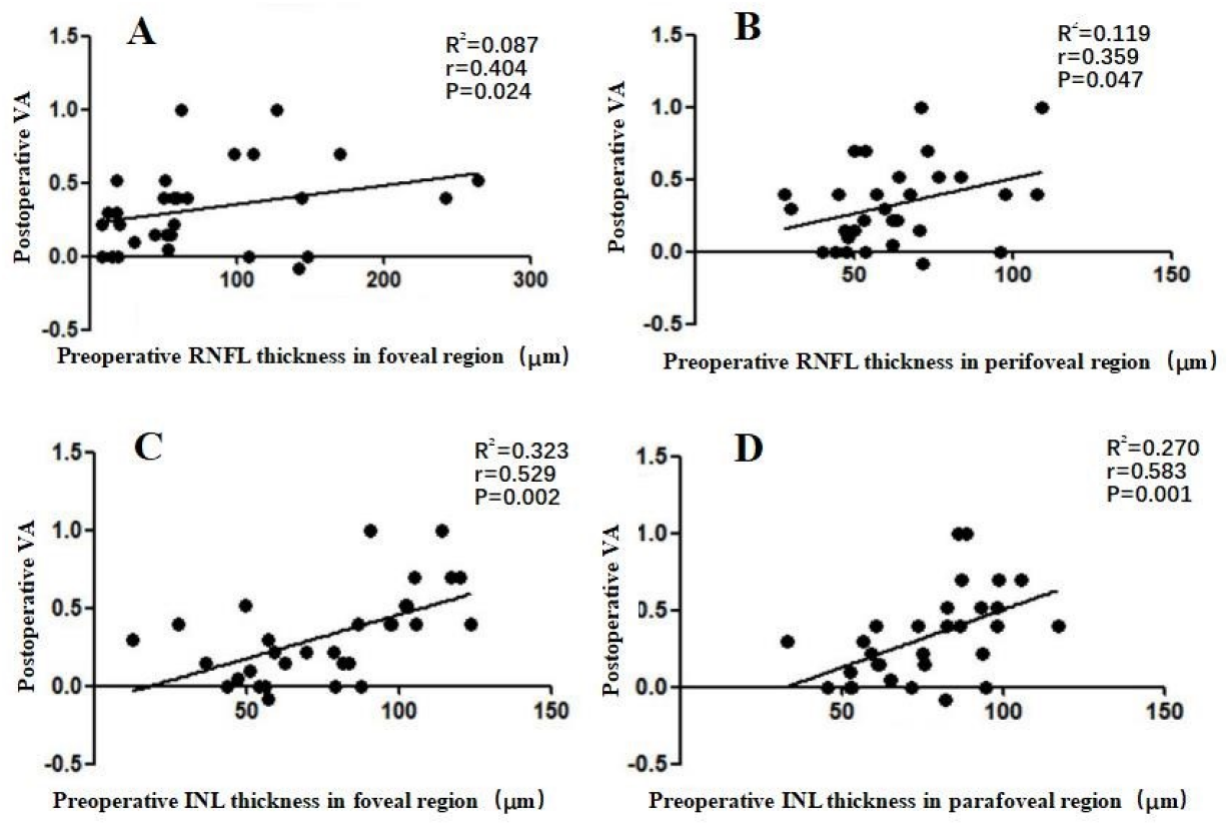
Correlation between postoperative VA at 6 months after an operation and preoperative individual retinal layer thickness in each macular region of 31 surgical patients. (A) The postoperative VA at 6 months was weakly correlated with the preoperative RNFL thickness in the foveal region (*r* = 0.404). (B) The postoperative VA at 6 months was weakly correlated with preoperative RNFL thickness in the perifoveal region (*r* = 0.359). (C) The postoperative VA at 6 months was moderately correlated with the preoperative INL thickness in foveal region (*r* = 0.529). (D) The postoperative VA at 6 months was moderately correlated with the preoperative INL thickness in the parafoveal region (*r* = 0.583). Abbreviations: RNFL, retina nerve fiber layer; INL, inner plexiform layer; VA, visual acuity.

We additionally evaluated the correlation between postoperative VA and preoperative retinal thickness. In the 31 surgical ERM patients, we found a weak correlation between postoperative VA and preoperative RNFL thickness in the foveal and perifoveal regions (*r* = 0.404 and 0.359, respectively; *P* ≤ 0.047), and a moderate correlation between postoperative VA and preoperative INL thickness in the foveal and parafoveal regions (*r* = 0.529 and 0.583, respectively; *P* ≤ 0.002) (Fig. 3).

**Figure 4:**
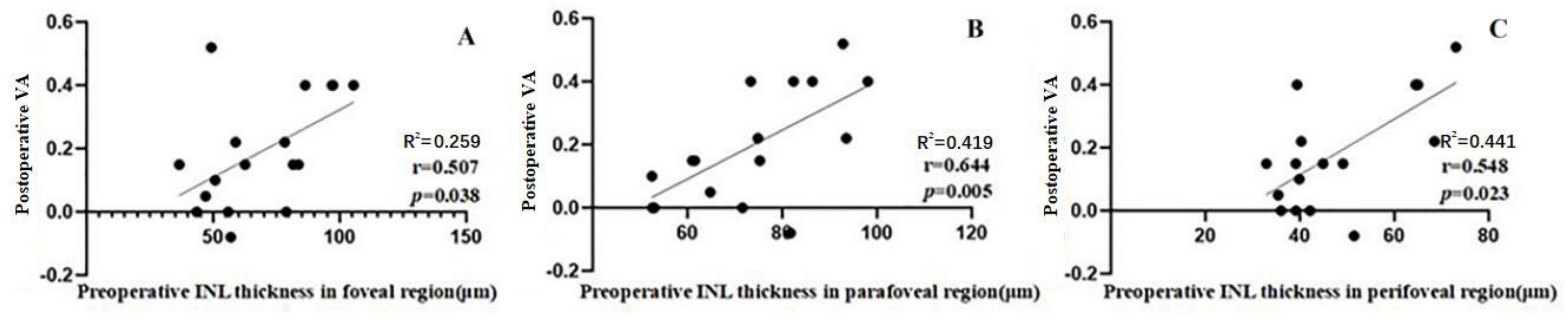
Correlation between postoperative VA at 6 months and the preoperative individual retinal layer thickness in each macular region of 17 patients whose VA gained ≥ 2 lines after an operation. (A) The postoperative VA at 6 months was moderately correlated with the preoperative INL thickness in the foveal region (*r* = 0.507). (B) The postoperative VA at 6 months was moderately correlated with preoperative INL thickness in the parafoveal region (*r* = 0.644). (C) The postoperative VA at 6 months was moderately correlated with the preoperative INL thickness in the perifoveal region (*r* = 0.548). Abbreviations: INL, inner plexiform layer; VA, visual acuity.

Seventeen of the 31 patients saw an improvement of more than two Snellen lines 6 months after surgery. In patients with visual improvement, we also detected a correlation between postoperative VA and the preoperative thicknesses of the seven individual retinal layers in each macular region. Only the INL thickness in the foveal, parafoveal, and perifoveal regions showed a correlation with the postoperative VA (*r* = 0.507, *P* = 0.038; *r* = 0.644, *P* = 0.005; *r* = 0.548, *P* = 0.023) (Fig. 4).

**Figure 5:**
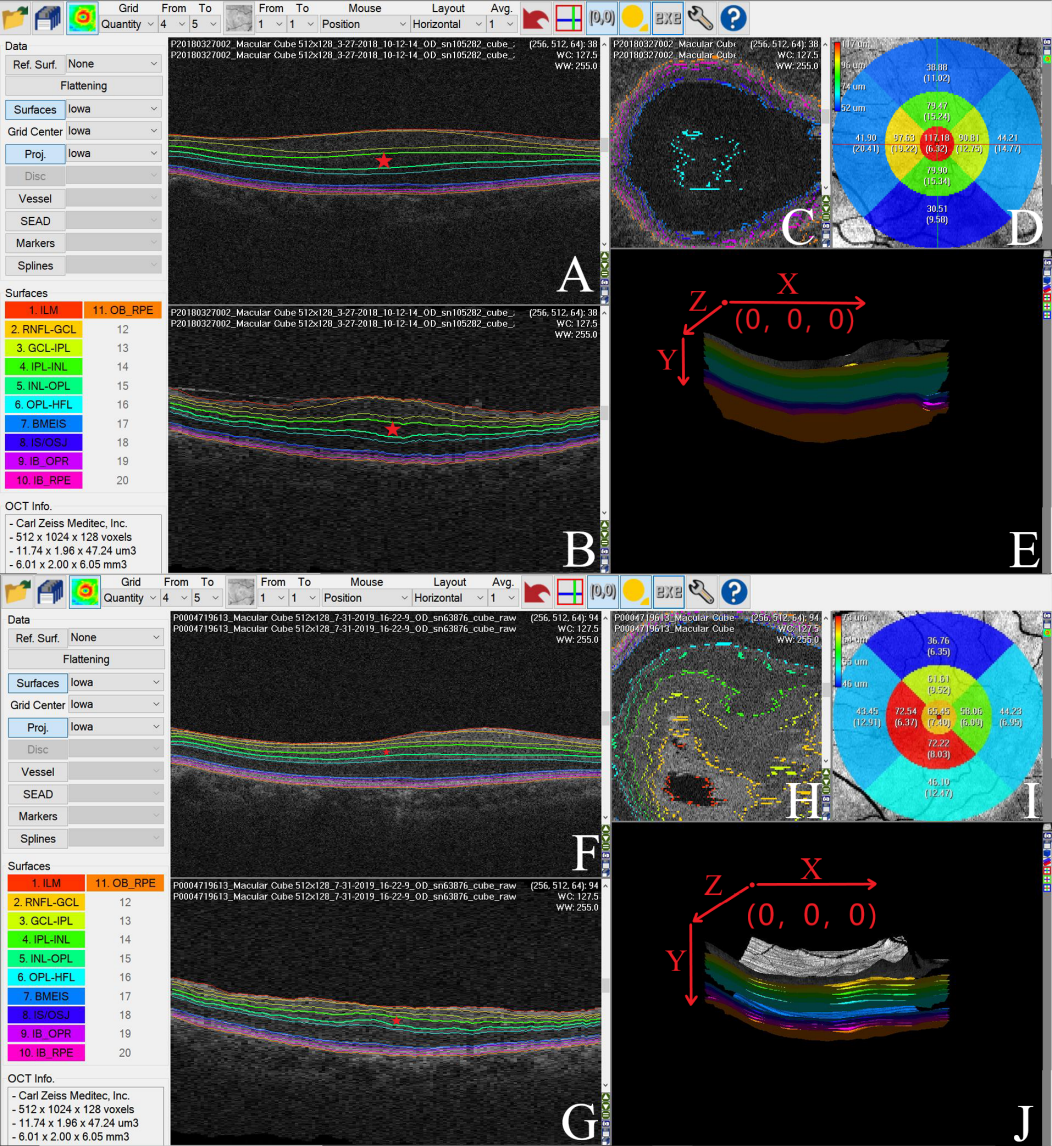
We used the Iowa Reference Algorithm to segment and measure the thickness of individual retinal layers in pre- and postoperative optical coherence tomography (OCT) images of one patient with ERM. The INL was significantly thinner following surgery. (A) Preoperative OCT XY image (B scan). The Iowa Reference Algorithm automatically segmented retinal boundaries in each OCT image, and the colored lines represent the interfaces between two adjacent retinal layers. The individual retinal layers were identified as follows (from the inner to outer surface): retinal nerve fiber layer (RNFL), ganglion cell layer (GCL), inner plexiform layer (IPL), inner nuclear layer (INL), outer plexiform layer (OPL), outer nuclear layer (ONL), photoreceptor inner/outer segments (IS/OS), outer segment (OS), and retinal pigment epithelium (RPE). The pentagram represents the INL in the OCT image. (B) Preoperative OCT ZY image. The colored lines represent the interfaces between two adjacent retinal layers, and the individual retinal layers were identified as follows (from the inner to outer surface): RNFL, GCL, IPL, INL, OPL, ONL, IS/OS, OS and RPE. The pentagram represents the INL in the OCT image. (C) Preoperative OCT XZ image. The colored lines represent the interfaces between two adjacent retinal layers. (D) The mean INL thickness in each subfield of the preoperative OCT image. (E) Preoperative OCT 3D image and its orientation (X, Y, and Z axial). (F) Postoperative OCT XY image (B scan). The colored lines represent the interfaces between two adjacent retinal layers, with the individual retinal layers identified as follows (from the inner to outer surface): RNFL, GCL, IPL, INL, OPL, ONL, IS/OS, OS, and RPE. The pentagram represents the INL in the OCT image. (G) Postoperative OCT ZY image, with the individual retinal layers identified as follows (from the inner to outer surface): RNFL, GCL, IPL, INL, OPL, ONL, IS/OS, OS, and RPE. The colored lines represent the interfaces between two adjacent retinal layers. The pentagram represents the INL in the OCT image. (H) Postoperative OCT XZ image, with the color lines representing the interfaces between two adjacent retinal layers. (I) The mean INL thickness in each subfield of the postoperative OCT image. (J) Postoperative OCT 3D image and its orientation (X, Y, and Z axial).

Figure 5 shows an automatic Iowa Reference Algorithm segmentation of pre- (A, B, C, and E) and postoperative (F, G, H, and J) retinal scanning where the INL became thinner following surgery (D and I).

## Discussion

We found significant correlations between VA and INL thickness in the foveal, parafoveal, and perifoveal regions of ERM eyes before and after surgery. Our results suggest that increased INL thickness in each macular region can lead to visual impairment, and that recovery of INL thickness may be a predictor of better postoperative visual outcomes.

Although metamorphopsia and visual impairment could be improved in approximately 70–90% of postoperative ERM cases (De Bustros et al., 1988; Iuliano et al., 2019), these symptoms persist in some ERM patients. Predicting postoperative outcomes and weighing the risks and benefits of surgery are important because there are currently no standardized surgical indicators for ERM (Diaz-Valverde & Wu, 2018; Nakashizuka et al., 2019). Ganglion cell complex thickness, outer retina thickness, length of photoreceptor segments (PROS), and INL thickness have been shown to change after surgery and may be prognostic factors for ERM patients (Okamoto et al., 2015; Pierro et al., 2015; Shiono et al., 2013; Takabatake et al., 2018). Previous studies have focused on the foveal retinal layers, ignoring the function of the parafoveal and perifoveal retinal layers. Kim et al. (2013) investigated the thickness of the inner layer in the superior, inferior, nasal, and temporal subfields at 500 µm and 600 µm from the foveal center, before and after surgery. They found that after surgery, it took longer for the inner layers of the subfields in the fovea and nasal parafovea to return to normal than the inner layers of the superior, inferior, and temporal macula. The results of this study suggested that evaluation of individual retinal layer thickness in different macular regions before and after surgery, is important for gaining a better understanding of ERM pathogenesis.

Manual segmentation is regarded as a flawed method for assessing individual retinal layer thickness in ERMs (Koo, Rhim & Lee, 2012; Okamoto et al., 2015). The Iowa Reference Algorithm is a powerful segmentation software that can be used to automatically segment retinal layers (Sohn et al., 2013; Song, Lee & Smiddy, 2016). Terry et al. (2016) recommended using the Iowa Reference Algorithm for healthy retinal segmentation. Sohn et al. (2013) showed its efficiency and stability by comparing its retinal thickness measurements to those of the Heidelberg Spectralis optical coherence tomography algorithm in eyes with diabetic macular edema. These studies demonstrated the remarkable repeatability and reliability of the Iowa Reference Algorithm. Additionally, the Iowa Reference Algorithm allows for automatic computation of individual retinal layer thickness in the nine regions of the retina according to the ETDRS grid. All these properties contribute to the accurate measurement of individual retinal layer thickness in each macular region (Demirkaya et al., 2013). In this study, all OCT scanning that met the criteria could be segmented successfully (Fig. 5).

Arichika, Hangai & Yoshimura (2010) proven that the inner, outer, and total retina thicknesses in eyes with ERMs were greater than those of normal eyes. We conducted a multiple linear regression analysis on the individual thicknesses in each macular region of 105 ERM patients. Our results showed that although the thicknesses of the foveal INL, foveal RNFL, parafoveal INL, parafoveal OPL, parafoveal PRL, and perifoveal INL may affect VA, changes in INL thickness might play the most essential role in the visual impairment of ERM patients. The *R*^2^ values of the three predicting models were 0.344, 0.427, and 0.340, respectively, indicating that the models could explain 34%–42.7% of the VA in ERM patients. The correlations between VA and INL thickness in the foveal, parafoveal, and perifoveal regions were 0.524, 0.572, and 0.626, respectively. These data indicated that there were other factors involved in the visual impairment of ERM patients. Previous studies reported that IS/OS integrity, cone outer segment tip integrity, and other retinal changes are potential factors impacting the VA of ERM patients (Garcia-Fernandez et al., 2013; Inoue et al., 2011; Kim et al., 2013). Therefore, VA and other retinal changes that may lead to visual impairment in ERM patients warrant further study.

Compared to the preoperative thickness in each macular region, there was a decrease in the total retinal thickness of each macular region. These results indicated that tractional force affects almost all layers in each of the retina’s macular regions. Anatomical damage to the macular retina may cause visual symptoms, resulting in decreased VA and increased macular retinal thickness. In the 31 surgical patients, we found significant decline 6 months postoperative in the thicknesses of the foveal RNFL, GCL, OPL, and PRL; the parafoveal GCL, OPL, and PRL; and the perifoveal ONL. This indicated that anatomical restoration following idiopathic ERM peeling was quickest in the perifoveal region, followed by the parafoveal region, and finally the foveal region. Nerve fibers, ganglion cells, horizontal cells, bipolar cells, and other cells associated with VA were more closely packed in the foveal region compared to the parafoveal and perifoveal regions, which may be the reason why foveal anatomical restoration was slower following surgery (Treumer et al., 2011). Additionally, other studies have found that 43% of eyes show visual improvement 12 months postoperative, with the percentage increasing over time (Pesin et al., 1991). This is most likely due to the retinal layers that recover gradually from tractional force after surgery, and decreasing macular retinal thickness in each region leading to improved VA.

Both postoperative VA and VA gain are important postoperative evaluation indexes. In our surgical ERM patients, we found that postoperative VA had a weak correlation with preoperative foveal and perifoveal RNFL thickness, but a moderate correlation with foveal and parafoveal INL thickness. However, only INL thickness in the foveal, parafoveal, and perifoveal regions correlated to VA restoration. Therefore, INL thickness in the foveal, parafoveal, and perifoveal regions may have prognostic value for postoperative VA gains in patients with ERM. Our results were consistent with those of previous studies that showed that foveal and parafoveal INL thickness were closely related to preoperative and postoperative VA and metamorphopsia scores in patients with ERMs (Kim et al., 2013; Okamoto et al., 2015; Zur et al., 2018). However, it remains unclear why perifoveal INL damage affects VA. Other researchers have investigated ERM patients’ dramatic blood flow velocity decrease in their perifoveal retina, as well as their increased blood flow after surgery (Coppe et al., 2019; Kadonosono et al., 1999; Kim et al., 2018; Li, Xia & Paulus, 2018). Kim et al. (2018) suggested that centripetal displacement and macular vascular integrity loss may lead to vascular insufficiency in the ERM, causing further neuronal deterioration. We speculate that the INL, composed of synaptic junctions, neuronal cells, and choroidal vessels, is more sensitive to idiopathic ERM-induced microvascular damage in ERM patients.

We acknowledge several limitations in this study. First, this was a retrospective study. Although we collected data from 105 ERM patients, the number of eligible surgical patients (31) was limited. Additional studies using more powerful analysis tools and larger sample sizes are needed. Second, Snellen charts are inappropriate for accurate clinical and research measures of VA (Lovie-Kitchin, 2015; Mataftsi et al., 2019). Although our pilot study does present preliminary results for larger prospective clinical validation, future clinical and research studies should use charts incorporating the Bailey-Lovie design principle and record VA in logMAR notation to get more precise measurements. Third, the ratio of visual improvement can change over time, and our postoperative follow-up time was only 6 months. Further research is needed to determine the relationship between the preoperative thickness of individual retinal layers in each macular region and postoperative VA over different periods of time following surgery. Finally, future studies should further examine macular retinal function, especially the function of the perifoveal retina in patients with ERMs.

## Conclusions

In conclusion, our results indicated that ERM affects almost all retinal layers in each macular region. We found a positive correlation between preoperative INL thickness in the foveal, parafoveal, and perifoveal region and preoperative VA and VA restoration. Therefore, foveal, parafoveal, and perifoveal INL thickness may be a marker for postoperative VA prognosis in patients with ERMs.

##  Supplemental Information

10.7717/peerj.9481/supp-1 Supplemental Information 1 Preoperative information of 105 patients

10.7717/peerj.9481/supp-2 Supplemental Information 2 31 patients followed up over 6 months and without progression of cataract after surgery

10.7717/peerj.9481/supp-3 Supplemental Information 3 Postoperative best-corrected visual acuity (BCVA) at 6 months and preoperative individual retinal layer thickness of 17 patients (BCVA gained ≥2 lines)

## Additional Information and Declarations

### Data Availability

The following information was supplied regarding data availability: The raw measurements are available in the Supplementary Files.
